# Reduced Size and Macrophage Content of Advanced Atherosclerotic Lesions in Mice with Bone Marrow Specific Deficiency of Alpha 7 Nicotinic Acetylcholine Receptor

**DOI:** 10.1371/journal.pone.0124584

**Published:** 2015-03-31

**Authors:** Robert H. Lee, Guillermo Vazquez

**Affiliations:** Department of Physiology and Pharmacology, Center for Diabetes and Endocrine Research, University of Toledo College of Medicine, Health Science Campus, 3000 Transverse Dr., Toledo, Ohio, 43614, United States of America; University of Toledo School of Medicine, UNITED STATES

## Abstract

In macrophages the α7 nicotinic acetylcholine receptor (α7nAChR) modulates production of inflammatory cytokines, cholesterol accumulation and lipoprotein uptake. Recently, our laboratory showed that selective stimulation of the α7nAChR protects macrophages from apoptosis, an effect that is absent in α7nAChR-deficient macrophages. All these observations are suggestive of a potential role of macrophage α7nAChR in atherosclerosis. Mouse models of the disease with bone marrow deletion of α7nAChR represent an attractive approach to address the *in vivo* relevance of these *in vitro* findings. However, recent studies that focused on the impact of hematopoietic deficiency of α7nAChR on early atherosclerotic lesions of low density lipoprotein receptor knockout (LDLRKO) mice, yielded controversial results. The question also remained whether macrophage α7nAChR modulates the characteristics of advanced lesions. Here we used LDLR knockout mice transplanted with bone marrow from wild-type or α7nAChR knockout animals to revisit the effect of hematopoietic deficiency of α7nAChR on early lesions and to examine, for the first time, its impact on advanced plaques. Aortic sinus atherosclerotic lesions were analyzed following 8 and 14 weeks on a high fat diet. Early lesions in mice with α7nAChR deficient bone marrow were not different from those in control animals. However, advanced lesions of mice with bone marrow deletion of α7nAChR exhibited reduction in size, macrophage content and cell proliferation. These studies are the first in examining the impact of hematopoietic deficiency of α7nAChR on the characteristics of advanced atherosclerotic lesions in a mouse model of the disease and provide novel evidence underscoring a potential pro-atherogenic role of macrophage α7nAChR.

## INTRODUCTION

The α7 nicotinic acetylcholine receptor (α7nAChR) is a ligand gated, non-selective cation channel with homopentameric arrangement which exhibits relatively high permeability to calcium compared to other nAChRs [1]. Besides its canonical localization and functions in the central and peripheral nervous systems, the α7nAChR is also expressed in non-neuronal cells including endothelial cells, lymphocytes and macrophages [2–5]. Indeed, compelling experimental evidence supports diverse functions of α7nAChR in several non-neuronal tissues and organ systems and associated physiopathological processes. For example, in a number of non-neuronal cells activation of the α7nAChR promotes cell survival and protects cells from apoptosis [2, 6, 7]. In macrophages, activation of α7nAChR has been shown to suppress pro-inflammatory cytokine production in models of sepsis and acute inflammation [8–10]. The potential anti-inflammatory role of macrophage α7nAChR was further examined in peritoneal macrophages derived from a mouse model of atherosclerosis with global deficiency of α7nAChR [11]. These *in vitro* studies indicated that α7nAChR may contribute to regulation of macrophage cholesterol metabolism and lipoprotein uptake [11], and although this is suggestive of a potential role of macrophage α7nAChR in atherogenesis, those findings were not validated by *in vivo* studies.

Macrophage apoptosis plays a critical role in atherosclerotic lesion development [12]. In recent work from our laboratory we specifically examined the impact of α7nAChR activation on endoplasmic reticulum (ER) stress-induced apoptosis of bone marrow derived macrophages differentiated *in vitro* to the “classical” M1 and “alternative” M2 types [4]. Our findings showed that under conditions of chronic ER stress α7nAChR stimulation protects macrophages from apoptosis, with this protective effect being absent in α7nAChR-deficient macrophages [4]. Despite the above mentioned studies and the well-established role of macrophages in the maladaptive inflammatory response that accompanies most stages of atherosclerosis, the question remained whether in the setting of atherosclerosis macrophage α7nAChR could impact the characteristics and/or progression of lesions. Two recent studies aimed at examining the characteristics of early atherosclerotic lesions in low density lipoprotein receptor knockout (LDLRKO) mice receiving α7nAChR-deficient bone marrow, yielded controversial results [13, 14]. Johansson et al. [13] reported that hematopoietic deficiency of α7nAChR was correlated with reduced size of early aortic root lesions, whereas Kooijman et al. [14], using the same experimental conditions, found no differences in lesion burden or complexity. Surprisingly, both studies were conducted on one single time point and focused on early lesions, leaving unanswered the question whether bone marrow deficiency of α7nAChR could influence the characteristics of advanced atherosclerotic lesions. In the present study we used LDLRKO mice transplanted with bone marrow from wild-type or α7nAChR knockout animals to revisit the impact of hematopoietic deficiency of α7nAChR on early lesions and to examine, for the first time, its effect on advanced stage plaques. The transplanted LDLRKO mice were maintained for 8 and 14 weeks on a high fat diet to promote development of early and advanced stage atherosclerosis, respectively, and the size, cellularity and complexity of lesions in the aortic root was evaluated. Notably, whereas no significant differences were found in the characteristics of early stage atherosclerotic lesions (8 weeks), advanced plaques (14 weeks) from mice with bone marrow deficiency of α7nAChR were significantly smaller and with reduced macrophage content than lesions in control animals. Although lesional necrosis area, collagen content and cap thickness were similar between groups, cell proliferation was reduced in advanced plaques from mice with α7nAChR deficient bone marrow. Our studies are the first showing a longitudinal evaluation of atherosclerotic lesions in mice with bone marrow deficiency of α7nAChR and demonstrate a pro-atherogenic impact of hematopoietic α7nAChR on advanced atherosclerotic plaques. These results point to α7nAChR as a novel molecular target of therapeutic potential in advanced stages of atherosclerosis.

## MATERIALS AND METHODS

### Experimental animals

All studies involving animals described in this work conform to the Guide for the Care and Use of Laboratory Animals published by the U.S. National Institutes of Health and have been approved by University of Toledo IACUC. C57BL/6 mice, the α7nAChRKO (B6.129S7-Chrna7^tm1Bay/J^) mice and LDLRKO (B6.129S7-Ldlr^tm1Her/J^) mice were obtained from Jackson Labs (Jackson Labs, ME) and colonies were maintained in our animal facility. Euthanasia was performed by intraperitoneal injection of sodium pentobarbital (150mg/kg) added to an anti-coagulant (heparin, 10 Units/ml).

### Bone marrow transplantation (BMT)

Essentially as we described in detail in ref. [15]. Briefly, recipient mice (LDLRKO females, 6-week-old, C57BL/6 background) were irradiated (10 Gy, 3 min; ^137^Cs-Gammacell 40 Exactor, Nordion Int. Inc.) and 4 hours later injected via tail vein with bone marrow cells (~5×10^6^ cells) from C57BL/6 (B6) or α7nAChRKO (α7KO, on C57BL/6 background) mice. Preparation of bone marrow cells from donors is described in detail in ref. [15]. To determine chimerism, PCR was performed on cDNA from peripheral blood cells for α7nAChR (for α7KO→LDLRKO mice) and on gDNA from peripheral blood cells for wild-type LDLR (for B6→LDLR mice). Primers used for α7nAChR were: F (5'—>3'): AAT TGG TGT GCA TGG TTT CT; R (5'—>3'): AGC CAA TGT AGA GCA GGT TG (Realtimeprimers.com, accession NM_007390). PCR for α7nAChR was run (35 cycles) under the conditions described in [4]. For LDLR, primer sequences and PCR cycling conditions were as recommended by Jackson Labs (Jackson Labs, ME).

### Determination of plasma cholesterol and triglycerides

After a 12 h fasting period blood was collected by submandibular vein puncture. Total plasma cholesterol and triglyceride concentrations were determined using Cholesterol-E and L-Type Triglyceride-M (Wako Chemicals USA, Inc.) following manufacturer’s instructions.

### Aortic root sectioning

Aortic root sections were prepared as we described in ref. [16]. Briefly, euthanized mice were perfused through the left ventricle with 4% paraformaldehyde followed by PBS. The heart was cut so that all three aortic valves were in the same geometric plane. The upper portion of the heart was embedded in O.C.T., frozen in the Peltier stage of the cryostat (Thermo Scientific R. Allan HM550 Cryostat) and processed for sectioning. Sections (10 μm) were collected onto Fisher Superfrost Plus-coated slides, starting from where aorta exits the ventricle and moving towards the aortic sinus over ~650–700 μm. Additional sections were collected at the end to be used as controls in immunostaining procedures. Lesion analysis and Oil Red O (ORO), hematoxylin and eosin (H&E) or trichrome stainings were as described in ref. [15]. Necrotic cores and cap thickness were evaluated as we describe in detail in ref. [15]. Staining of sections for *in situ* TUNEL was performed using an *in situ* cell death detection kit (Roche, IN) as described in detail in refs.[17, 18], with the modifications described in ref. [15].

### Immunohistochemistry

Immunohistochemistry (IHC) was essentially as we described in refs. [15, 16]. Briefly, sections were fixed in acetone and processed for immunostaining for MOMA-2 (#sc-59332, Santa Cruz Biotechnology, TX) followed by incubation with biotinylated rabbit anti-rat antibody (Dako). After treatment with secondary antibodies sections were incubated with alkaline phosphatase-conjugated streptavidin (Dako). Counterstaining was with hematoxylin. Negative controls were performed by substituting the primary antibody with non-immune IgG from the same species and at the same concentration. Under these conditions, nonspecific immunostaining was not detected. Stained areas were captured (Micropublisher 3.3 Megapixel Cooled CCD Color Digital Camera) and measured (NIS Elements D). Either MOMA-2 or the rabbit polyclonal antibody to AIA31240 (Accurate Chemical and Science Corp.) was used to identify macrophages in co-localization immunofluorescence staining. Secondary antibody was Alexa Fluor-488 goat anti-rabbit (#A11008, Invitrogen, for AIA31240) or Alexa Fluor-488 anti-rat (#4416, Cell Signaling, for MOMA-2). Immunostaining for the Ki67 antigen was performed by incubating acetone-fixed frozen sections with an anti-Ki67 antibody (#ab66155, Abcam; 1:100 dilution) overnight at 4°C followed by anti-rabbit IgG Alexa Fluor-555 (#4413, Cell Signaling) at 1:1,000 dilution for 1 h.

### In situ immunostaining for M1 and M2 macrophages

M1 and M2 macrophages in lesions were identified by co-staining for iNOS (Abcam, #ab15323) or mannose receptor (#HM1049, Hycult Biotech), respectively, and macrophage (Accurate Chemical, #AIA31240), as follows: frozen sections were fixed in acetone (15 min, 4°C) followed by incubation with iNOS antibody (1:100 dilution) overnight at 4°C and then anti-rabbit IgG Alexa Fluor-555 (#4413, Cell Signaling) at 1:1,000 dilution for 1 h, or with mannose receptor antibody (1:100 dilution) overnight at 4°C and then anti-rat IgG Alexa Fluor-555 (#4417, Cell Signaling) at 1:1,000 dilution for 1 h. Next, AIA31240 antibody was used at a 1:100 dilution followed by staining with anti-rabbit IgG Alexa Fluor-488 (#A11008, Invitrogen) at 1:1,000 dilution for 1 h. Slides were mounted using the Prolong Gold Antifade Reagent with DAPI (Cell Signaling, #8961). All antibody dilutions were done using the IHC TEK antibody diluent (IW-1,000) from IHC World.

### Statistical analysis

Values are shown as mean ± SEM. Comparisons between groups was performed by Mann-Whitney U-test using Prism Graph Pad version 6 for Windows 7 (San Diego, CA). P values below 0.05 were considered significant.

## RESULTS

In this study we examined the effects of macrophage deficiency of α7nAChR on the characteristics of early and advanced stage atherosclerotic lesions in low density lipoprotein receptor knockout (LDLRKO) mice. To that end, we utilized a bone marrow transplantation approach, a strategy used successfully by our laboratory and others to study the contribution of a particular gene expressed by macrophages to atherogenesis [15, 19].

**Fig 1:**
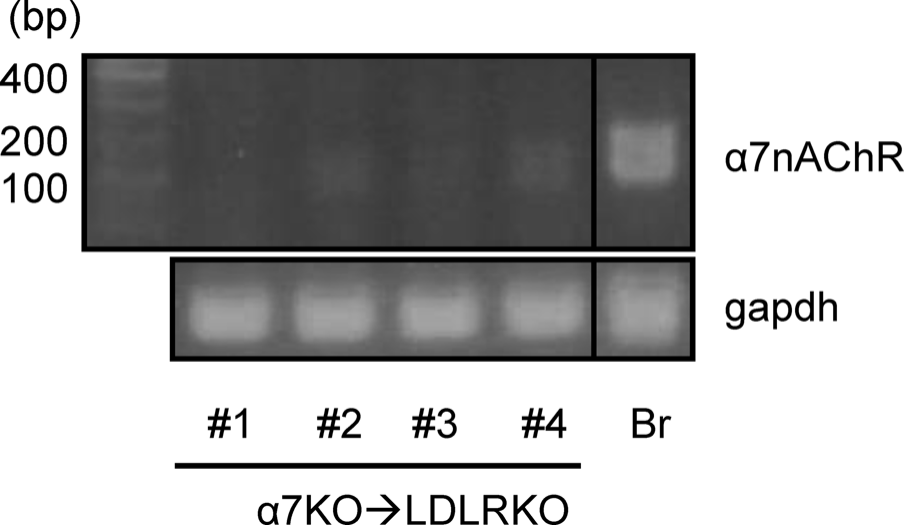
Phenotype conversion of mice transplanted with α7nAChRKO deficient bone marrow. Lethally irradiated female LDLRKO mice were transplanted with bone marrow from wild-type (B6→LDLRKO) or α7nAChRKO mice (α7KO→LDLRKO). Shown are examples of PCR performed on cDNA derived from pheripheral blood cells (submandibular vein puncture) from 4 α7KO→LDLRKO mice performed at four weeks after bone marrow transplantation to examine expression of α7nAChR. The 157 bp band corresponds to wild-type α7nAChR. Chimerism is evidenced by lack or low intensity band for the α7nAChR amplicon in the α7KO→LDLRKO group. cDNA from B6 brain (Br) was used as a positive control. B6→LDLR chimerism was determined by PCR on peripheral blood cell gDNA for wild-type LDLR (not shown; see methods).

Six week old female LDLRKO mice were lethally irradiated and transplanted with bone marrow from C57BL/6 (B6) or α7nAChRKO (α7KO) mice to create chimeric mice with either α7nAChR expressing (B6→LDLRKO) or deficient (α7KO→LDLRKO) bone marrow. Conversion to the desired phenotype was confirmed four weeks after transplantation by PCR of cDNA from peripheral blood (Fig 1). At this time, mice were placed on a Western type high fat diet (HFD; TD.88137, Harlan Teklad) for 8 or 14 weeks.

At sacrifice, we measured body weight, plasma cholesterol and triglycerides. As shown in Table 1, after 8 weeks on HFD body weights were comparable between both groups of mice. The α7KO→LDLRKO group had lower total plasma cholesterol levels compared to the control group; however, both groups were still markedly hypercholesterolemic (Table 1). There was no difference in total levels of plasma triglycerides after 8 weeks on HFD.

**Table 1:** Body weight and lipid profile for B6→LDLRKO vs. α7KO→LDLRKO mice.

	B6→LDLRKO	α7KO→LDLRKO	P value
Total cholesterol (mg/dl)	953 ± 211 (n = 6)	746 ± 94 (n = 7)	0.035
	1,063 ± 264 (n = 6)	1,158 ± 146 (n = 6)	n.s.
Triglycerides (mg/dl)	104 ± 25 (n = 6)	151 ± 106 (n = 7)	n.s.
	101 ± 39 (n = 6)	251 ± 111 (n = 6)	0.026
Body weight after diet (g)	21.0 ± 2.9 (n = 13)	21.2 ± 1.6 (n = 15)	n.s.
	22.1 ± 2.4 (n = 13)	23.4 ± 3.3 (n = 10)	n.s.

Mice were fed a high fat diet for 8 (first row of values for each parameter) and 14 weeks (second row of values for each parameter) and body weight and total plasma cholesterol and triglycerides were measured as described in Methods.

Values are mean ± SEM, for the indicated number of mice (in parenthesis). P values were obtained with the Mann-Whitney U-test; n.s.: not statistically significant.

**Fig 2:**
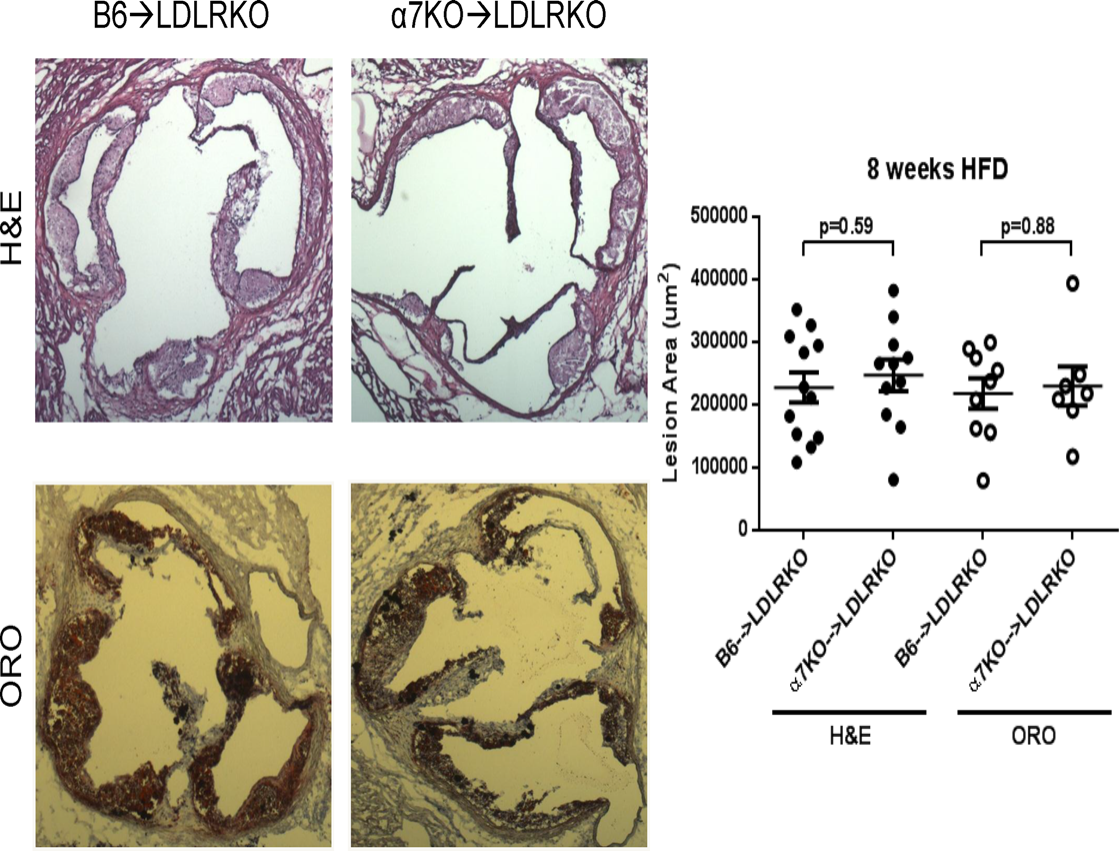
Size and lipid content of early stage aortic root lesions. Aortic root sections from B6→LDLRKO or α7KO→LDLRKO mice on an 8 week high fat diet were stained with hematoxylin-eosin (H&E) or Oil-Red-O (ORO) to evaluate lesion area and neutral lipid content, respectively. Quantitation of mean stained areas is shown. Mean values and corresponding standard errors and n numbers are provided in the text. P values were determined using the Mann-Whitney U-test.

To examine the impact of bone marrow deficiency of α7nAChR on lesion development we performed morphometric analysis on hematoxilin-eosin (H&E)-stained aortic root sections. After 8 weeks on HFD no differences were observed in total lesion area between the two groups of animals (227,219 ± 24,166 μm^2^ (n = 12) vs. 246,792 ± 25,201 μm^2^ (n = 11), for B6→LDLRKO vs. α7KO→LDLRKO, respectively; p = 0.59; representative sections are shown in Fig 2). Neutral lipid content, as assessed by Oil Red O (ORO) staining, was not different either (217,816 ± 24,450 μm^2^ (n = 9) vs. 229,553 ± 31,578 μm^2^ (n = 7), for B6→LDLRKO vs. α7KO→LDLRKO, respectively; p = 0.88, Fig 2). In addition, macrophage content was not significantly different between the two groups (MOMA2-positive areas: 151,345 ± 19,266 μm^2^ (n = 8) vs. 117,110 ± 20,518 μm^2^ (n = 7), for B6→LDLRKO vs. α7KO→LDLRKO, respectively; p = 0.86). The significant overlap between ORO and MOMA2 positive areas is in agreement with the notion of early lesions being dominated, for the most part, by foam cells. At this stage, acellular necrotic areas were not observed.

**Fig 3:**
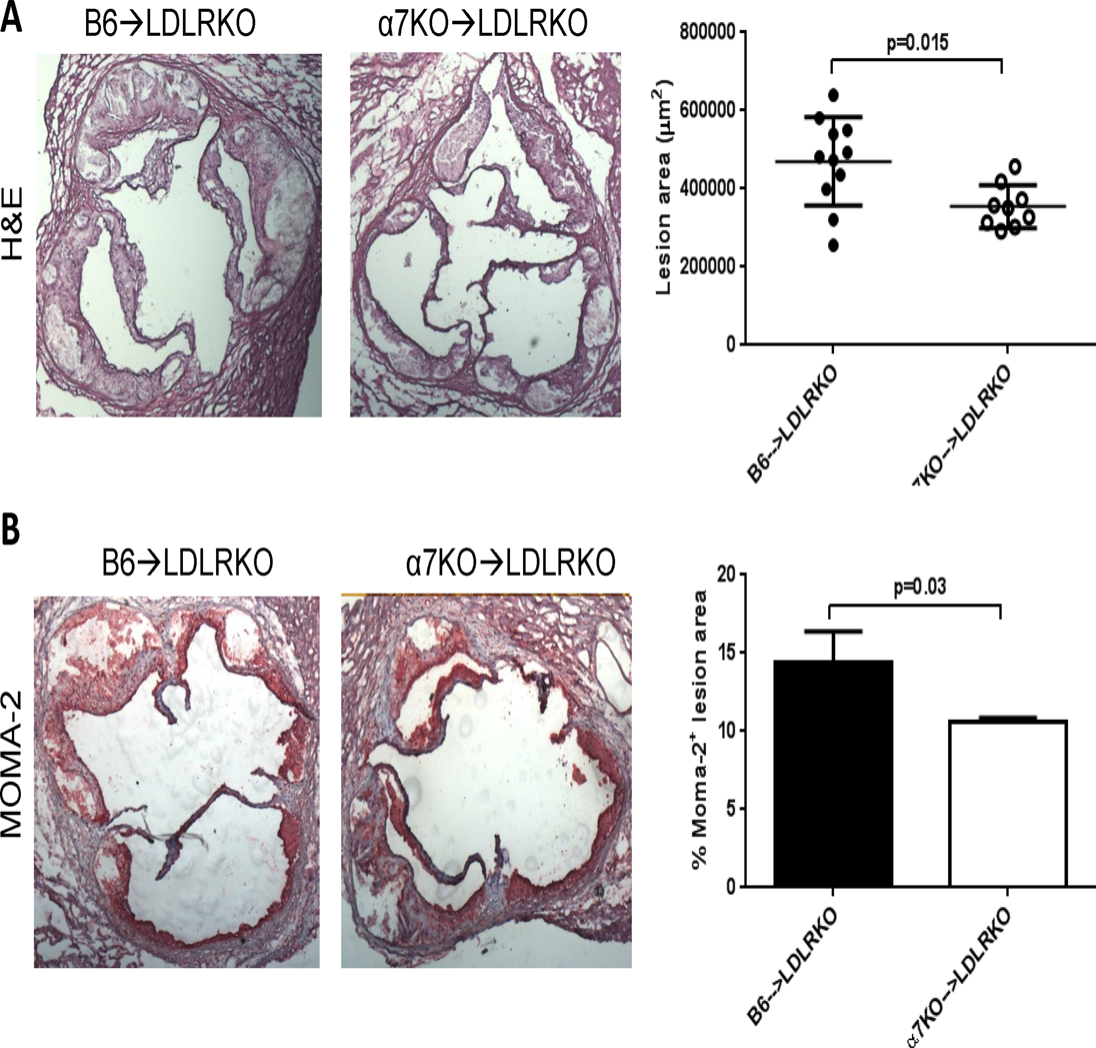
Size and macrophage content of advanced aortic root lesions. Atherosclerotic lesions in aortic root sections from LDLRKO mice transplanted with bone marrow from wild-type (B6→LDLRKO) or α7nAChRKO mice (α7KO→LDLRKO) that were maintained on a 14 week high fat diet, were stained with **A)** hematoxilin-eosin (H&E) to determine lesion area or **B)** MOMA-2 antibody to evaluate macrophage content. Shown are representative sections of H&E and MOMA-2 stainings, and corresponding quantitations of the mean stained areas. P values were determined using the Mann-Whitney U-test. In **A)** n = 11 and n = 9, in **B)** n = 6 and n = 5, for B6→LDLRKO and α7KO→LDLRKO, respectively.

We next examined aortic root lesions in mice maintained on HFD for 14 weeks. At time of sacrifice, body weights were similar between groups and there was no difference in total plasma cholesterol; however, the α7KO→LDLRKO mice had higher plasma triglycerides compared to the control group (Table 1). As expected, lesions at this stage were significantly bigger than those observed after 8 weeks on HFD and lesions with acellular, necrotic areas were clearly identified. As shown in Fig 3A, morphometric analysis of these lesions showed a reduction in total lesion area in α7KO→LDLRKO mice compared to control animals (354,672 ± 18,246 μm^2^ (n = 9) vs. 469,927 ± 34,028 μm^2^ (n = 11), for α7KO→LDLRKO vs. B6→LDLRKO, respectively; p = 0.015). At this time, macrophage content of the lesions was also markedly decreased in mice with bone marrow deficiency of α7nAChR (MOMA2-positive area as percent of total lesion area: 10.6 ± 0.3% (n = 5) vs. 14.4 ± 2.0% (n = 6), for α7KO→LDLRKO vs. B6→LDLRKO, respectively; p = 0.03, Fig 3B). The number of total (CD11b^+^) or inflammatory (Ly6C^high^) circulating monocytes were not different between B6→LDLRKO vs. α7KO→LDLRKO mice (not shown). The percent necrotic core area between groups was not different (27 ± 2 (n = 11) vs. 30 ± 2 (n = 9) %, for B6→LDLRKO vs. α7KO→LDLRKO, respectively; p = 0.37). Despite no statistically significant differences between groups in collagen content or cap thickness there was a consistent trend for a reduction in both parameters in α7KO→LDLRKO compared to control animals (normalized collagen content: 3.45 ± 0.28 vs. 2.79 ± 0.20, respectively, p = 0.12; fibrous cap thickness: 48.9 ± 6.4 μm (n = 10) vs. 36.9 ± 3.4 mm (n = 8), respectively, p = 0.23). Immunostaining for the T-cell marker CD3 did not show any differences between groups (69 ± 18 vs. 91 ± 18 CD3^+^ cells/mm^2^, for B6→LDLRKO vs. α7KO→LDLRKO, respectively; n = 4 for both groups, p = 0.48).

**Fig 4:**
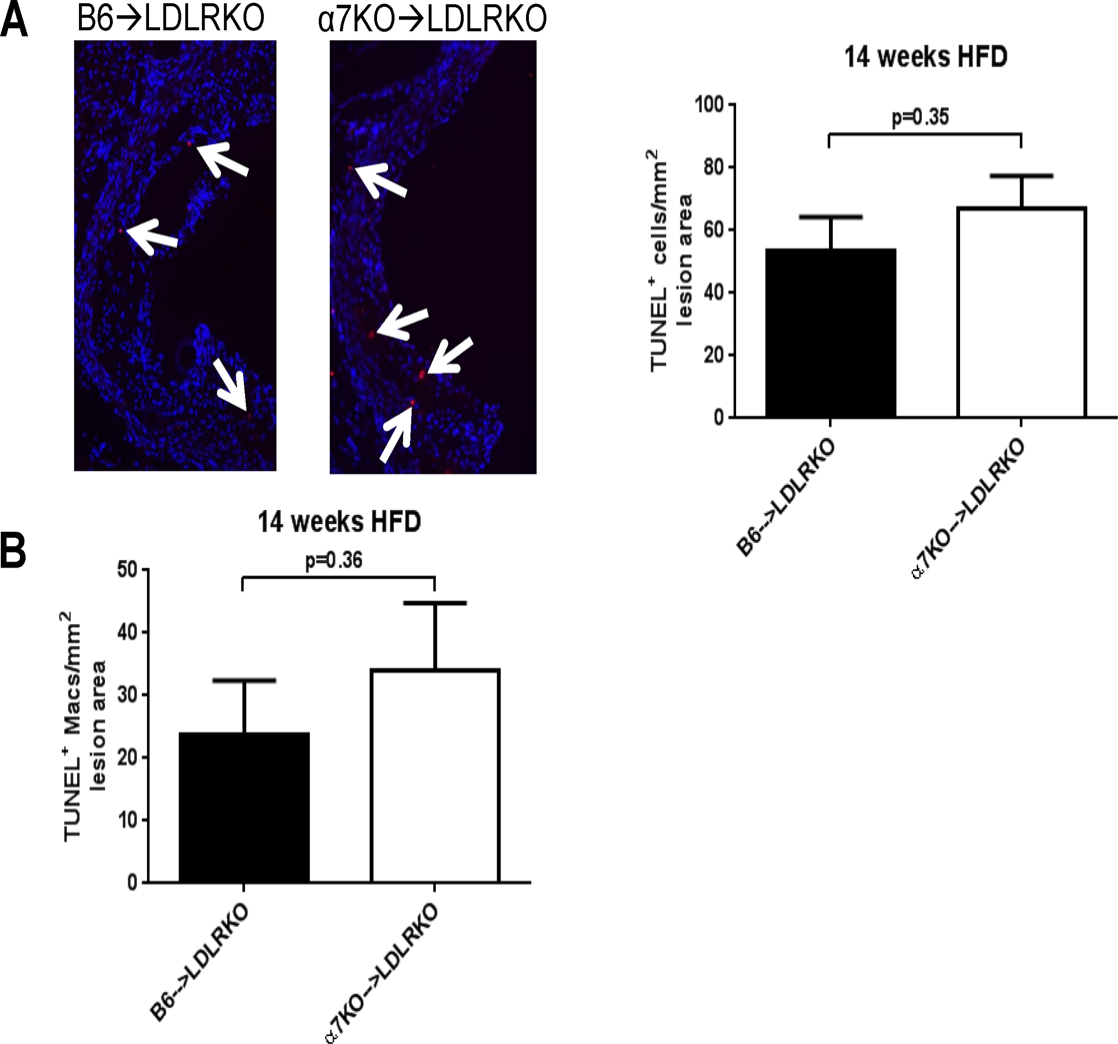
Apoptotic cells and macrophages in advanced aortic root lesions. Aortic root sections from LDLRKO mice transplanted with bone marrow from wild-type (B6→LDLRKO) or α7nAChRKO mice (α7KO→LDLRKO) that were maintained on a 14 week high fat diet, were stained for **A)** *in situ* TUNEL to detect apoptotic cells, or **B)** co-stained for macrophage (AIA31240) and TUNEL to evaluate apoptotic macrophages (as we described in [15]). The number of TUNEL^+^ (**“A”**) or AIA31240^+^ + TUNEL^+^ (**“B”**) cells were counted throughout the aortic sinus and normalized to the total lesion area. Representative stained sections are shown in **“A”**. Bar graphs show results as mean ± SEM. In both **“A”** and **“B”** n = 7 and n = 6, for B6→LDLRKO and α7KO→LDLRKO, respectively. P values were determined using the Mann-Whitney U-test.

In advanced atherosclerotic plaques, macrophage apoptosis is notorious and plays a key role in necrotic core growth and plaque stability [12]. Using a modified *in situ* TUNEL technique (described in detail by us in ref. [15]) we examined if bone marrow deficiency of α7nAChR affected accumulation of apoptotic cells in mice maintained on HFD for 14 weeks. As shown in Fig 4A, the number of total apoptotic (TUNEL^+^) cells was similar between groups after 14 weeks on HFD regardless of the α7nAChR expression status of the donor’s bone marrow. Interestingly, although not reaching statistical significance, there was a consistent trend for increased number of apoptotic macrophages in lesions from α7KO→LDLRKO mice compared with control animals, as evidenced by the co-localization of TUNEL^+^ cells and macrophage staining (Fig 4B). In a recent study we found that, *in vitro*, selective stimulation of macrophage α7nAChR results in a protective effect against ER stress-induced apoptosis, and this was rather selective for M2 macrophages compared to M1 cells [4]. To determine if in the setting of atherosclerosis the number of apoptotic macrophages was being influenced by an effect of bone marrow deficiency of α7nAChR on the abundance of lesional M2 macrophages, we examined by immunofluorescence the co-localization of mannose receptor, an M2-associated marker [20], with macrophage staining. Whereas significant mannose receptor staining co-localized with macrophage immunoreactivity (AIA31240^+^ cells), there were no differences in M2 macrophage content relative to lesion area between B6→LDLRKO and α7KO→LDLRKO mice (50.2 ± 13.7 vs. 71.3 ± 20.6 M2 cells/mm^2^, for B6→LDLRKO vs. α7KO→LDLRKO, respectively; n = 5 for both groups, p = 0.39). The number of M1 macrophages in advanced lesions from both groups of mice, as evaluated from the co-localization of iNOS, an M1 marker [21], with macrophage staining was not different either (not shown).

**Fig 5:**
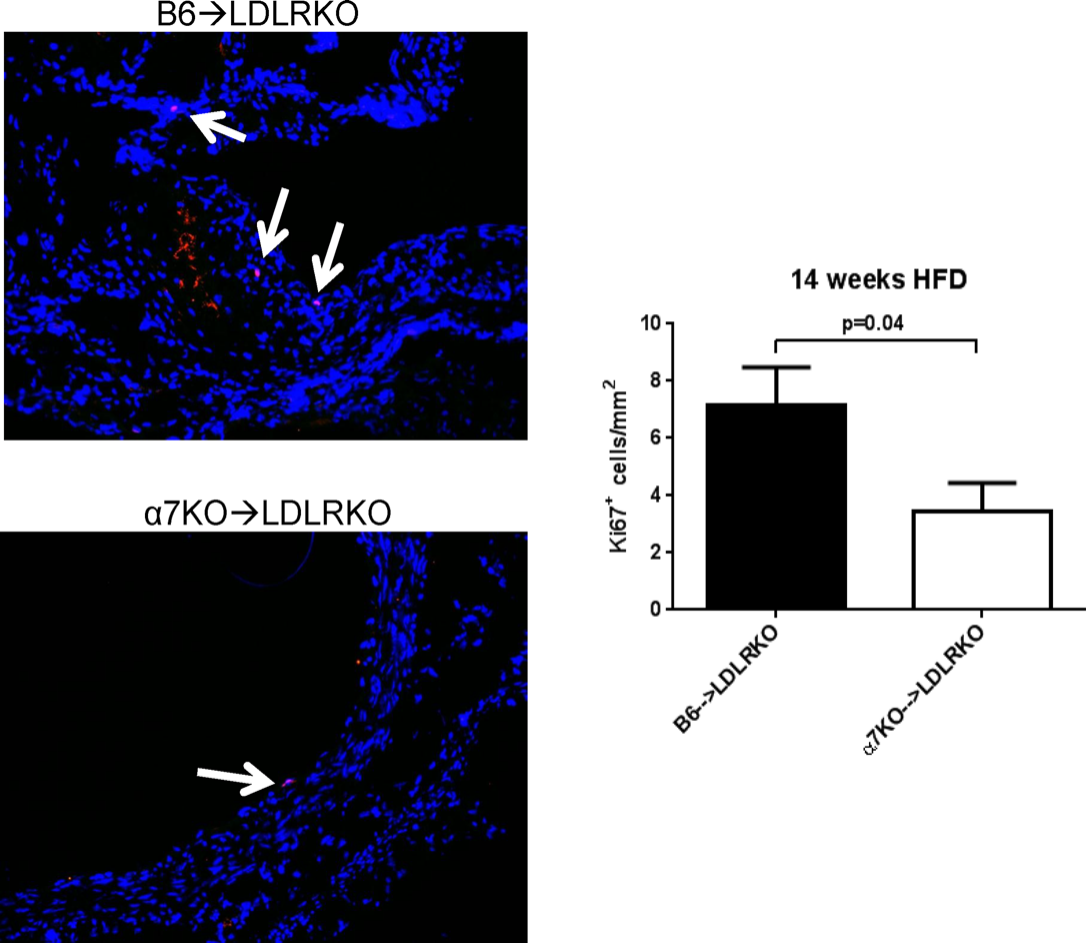
Cell proliferation in advanced aortic root lesions. Aortic root sections from LDLRKO mice transplanted with bone marrow from wild-type (B6→LDLRKO) or α7nAChRKO mice (α7KO→LDLRKO) and maintained on a 14 week high fat diet, were stained for the Ki67 antigen (red) to detect proliferating cells, and DAPI (nuclei). The arrows show the localization of Ki67 positive cells inside lesion areas. The number of Ki67^+^ cells were counted throughout the aortic sinus and normalized by the total lesion area. Results are mean ± SEM; n = 6 for both groups. P values were determined using the Mann-Whitney U-test. Magnification: x20.

Recent evidence indicates that the macrophage population of advanced atherosclerotic lesions is maintained mostly through proliferation of lesional macrophages rather than through newly recruited monocytes [22]. To examine if bone marrow deficiency of α7nAChR had an impact on the proliferative status of cells in lesions from mice maintained on 14-week HFD, aortic root sections from B6→LDLRKO and α7KO→LDLRKO mice were subjected to immunostaining for the nuclear antigen Ki67 (Fig 5). Notably, lesions from mice with α7nAChRKO bone marrow showed a reduced number of Ki67-positive cells compared to lesions in control animals (7.1 ± 1.3 vs. 3.4 ± 0.9 Ki67^+^ cells/mm^2^, for B6→LDLRKO vs. α7KO→LDLRKO, respectively; n = 8 for both groups, p = 0.04). In addition, co-localization of Ki67-positive cells with macrophage immunoreactivity exhibited a marked trend for reduction in mice that received α7nAChRKO bone marrow compared with controls (5.0 ± 0.8 vs. 2.7 ± 0.6 Ki67^+^ cells/mm^2^, for B6→LDLRKO vs. α7KO→LDLRKO, respectively; n = 8 for both groups, p = 0.08).

## DISCUSSION

The present studies represent the first longitudinal evaluation of the impact of bone marrow deficiency of α7nAChR on the morphometric characteristics, cellularity and complexity of both early and advanced atherosclerotic lesions in the aortic sinus of a mouse model of the disease. Whereas early stage lesions in LDLRKO mice with α7nAChR deficient bone marrow were not different from those in control animals, bone marrow deficiency of α7nAChR had a clear favorable effect on advanced stages, as evidenced by a marked reduction in size, macrophage content and cell proliferation in lesions of mice maintained on a fourteen week high fat diet.

Evidence accumulated over recent years underscored novel physiopathological roles of α7nAChR in a number of non-neuronal cell types, including macrophages. Studies in mouse models of sepsis and acute inflammation first suggested a potent anti-inflammatory role of macrophage α7nAChR [8–10]. Nevertheless, this novel function of α7nAChR seemed rather specific to those pathologies, with the receptor working as part of the so called cholinergic anti-inflammatory circuit. In an attempt to examine some of the functions of macrophage α7nAChR that might be of relevance to atherosclerosis Wilund et al. [11] studied peritoneal macrophages derived from ApoE knockout mice with global deficiency of α7nAChR and found that these cells exhibited increased cholesterol accumulation and oxidized LDL uptake compared to control cells [11]. Whereas these two processes are of significance to the *in vivo* functions of lesional macrophages during atherosclerosis, the *in vivo* relevance of these *in vitro* findings was not examined. Two groups have recently reported their findings on the effects of hematopoietic deficiency of α7nAChR in early atherosclerosis. Johansson et al. showed that male LDLRKO mice receiving α7nAChR-deficient bone marrow had a marked reduction in the size of early aortic root lesions after an eight week high fat diet schedule, but no differences were observed in lesion cellularity or complexity [13]. On the contrary, Kooijman et al. found no differences in size or composition of early lesions in female LDLRKO mice with bone marrow deficiency of α7nAChR following seven weeks on high fat diet [14], despite an increased inflammatory status of the α7nAChR-deficient mice. Our results showing that early lesions of female LDLRKO mice have similar size and content of foam cells regardless of the expression status of α7nAChR in the bone marrow, are in agreement with the findings of Kooijman et al. [14] and indicate that in the inflammatory setting of atherosclerosis macrophage α7nAChR does not have a major role in regulation of lipid handling and/or accumulation. Interestingly, in our studies and those by Kooijman et al. [14] female LDLRKO mice were used as bone marrow recipients, whereas male LDLRKO mice were used in the studies by Johansson et al. [13]. Whether the discrepant observations by the latter are to some extent related to gender-based differences in the effects of hematopoietic α7nAChR on early lesion characteristics remains to be determined.

In some non-neuronal cells such as T lymphocytes and endothelial cells activation of the α7nAChR promotes cell survival and protects cells from apoptosis [2, 6, 7]. Endoplasmic reticulum (ER) stress-induced macrophage apoptosis exerts a determinant role in progression and fate of atherosclerotic plaques [23]. Whereas in early lesions macrophage apoptosis is compensated by efficient clearance of apoptotic cells—efferocytosis- and thus slows lesion cellularity, in advanced plaques when efferocytosis is impaired apoptotic cells accumulate leading to enlargement of necrotic areas, a significant contributor to plaque instability. In recent work [4] we specifically examined the impact of macrophage deficiency of α7nAChR on ER stress-induced apoptosis of bone marrow derived macrophages differentiated *in vitro* to the M1 and M2 types, which better recapitulate, *in vitro*, the characteristics of *in vivo* macrophage populations compared to those of non-polarized macrophages. Our findings showed that under conditions of chronic ER stress selective α7nAChR stimulation protected macrophages from apoptosis [4]. Notably, this protective effect was absent in α7nAChR-deficient macrophages [4]. However, it was not evident from these observations whether in the setting of atherosclerosis macrophage α7nAChR could impact the characteristics and/or progression of lesions. The findings in the present studies indicate that in advanced lesions of mice with bone marrow deficiency of α7nAChR the total number of apoptotic macrophages shows a clear trend towards reduction, without significant changes in the number of M2 type macrophages. In addition, the necrosis content, collagen abundance and cap thickness of advanced plaques of LDLRKO mice were similar regardless of the bone marrow expression status of α7nAChR. *A priori* this may be interpreted as the α7nAChR protective actions on macrophages being of minor significance in the lesion setting, and that macrophage α7nAChR might not be a determining factor in mechanisms underlying necrotic core growth; however, the extent of activation of α7nAChR in lesional macrophages is uncertain. This is important considering that in our previous *in vitro* studies the protective actions of α7nAChR on M2 macrophages were evident upon stimulation of the receptor with a α7nAChR selective agonist. Therefore, future studies should examine whether stimulation *in vivo* of macrophage α7nAChR reveals functions that may have a physiopathological impact. Spleen T cells are capable of producing acetylcholine, and this has been proposed to be a source of acetylcholine responsible of α7nAChR activation in spleen macrophages [24]. Cholinergic innervation in coronary arteries from human and other mammals is sparse [25, 26], and one can speculate that the diffusibility and penetration of nerve terminal-derived acetylcholine into atherosclerotic plaques may be limited. In addition, neither M1 nor M2 macrophages seem to express the enzymatic machinery required for acetylcholine synthesis (Lee and Vazquez, unpublished). All this raises the question of how cholinergic signaling could modulate macrophage function in the distinctive setting of the atherosclerotic lesion. Whether a mechanism similar to that in spleen operates in the lesion microenvironment awaits elucidation of the capability of lesional cells to produce and locally release effective concentrations of acetylcholine.

As mentioned above, accumulation of apoptotic macrophages becomes more evident in intermediate and advanced lesions mostly due to progressive impairment of the efferocytic functions of resident phagocytes [27, 28]. In previous studies from our group we reported that deficiency of the non-selective cation channel Transient Receptor Potential Canonical 3 (TRPC3) in both M1 and M2 macrophages resulted, *in vitro*, in higher efferocytic capacity compared to Trpc3^+/+^ cells [15]. It thus remains to be determined if the efferocytic properties of α7nAChR deficient macrophages are altered and if so, to what extent this may compensate, *in vivo*, for any effects that α7nAChR deficiency may cause on the accumulation of apoptotic cells.

A remarkable observation derived from the present studies is that advanced lesions from mice with α7nAChR deficient bone marrow show a significant reduction in total lesion area and macrophage content. This is improbable to be related to differences in lipid metabolism as both groups were markedly hypercholesterolemic and the knockout group had even greater plasma triglycerides compared with controls. The reduced macrophage infiltration is unlikely to be due to changes in adhesion of circulating monocytes and/or the recruitment process as no differences in lesion size or macrophage content were found in early stage lesions, where the influx of monocytes is critical in determining lesion size and cellularity. In a recent study Robbins *et al*. [22] revisited the mechanism of macrophage accumulation during atherosclerotic lesion formation in a mouse model of the disease, and found that in advanced plaques proliferation of lesional macrophages, rather than continuous monocyte influx, accounts for a significant proportion of the macrophage content in the lesion [22]. Our immunofluorescence studies on advanced aortic root lesions showing a reduction in the number of total proliferating cells and that of macrophages co-localizing with the nuclear antigen Ki67 in mice with bone marrow deficiency of α7nAChR, suggest that reduced local proliferation can contribute to the decreased cellularity and size of the plaques in this group of mice compared with control animals. Indeed, because the most notorious phenotypic impact of bone marrow deficiency of α7nAChR was on the proliferation of lesional cells, this can in part explain the lack of significant effect on the early lesions, as at that stage the major determinant of lesion cellularity is the recruitment of circulating monocytes rather than proliferation of lesional macrophages [22]. The present findings represent the first *in vivo* evidence suggesting a pro-atherogenic role of macrophage α7nAChR in the advanced lesion setting. Notably, previous studies have shown that in both LDLRKO and ApoeKO mice, nicotine accelerates development of atherosclerosis through a mechanism that involves, to a great extent, direct actions on lesional macrophages [29, 30]. However, the specific receptor mediating this pro-atherogenic effect remains elusive. Our studies showing a positive correlation between bone marrow deficiency of α7nAChR and a reduction in size, cellularity and macrophage proliferation of advanced atherosclerotic lesions, point to α7nAChR as an interesting candidate receptor to mediate the pro-atherogenic actions of nicotine *in vivo*. Additional experimental work is required to identify which pro-atherogenic mechanisms are modulated by α7nAChR. Importantly, the present studies as well as those by Johansson et al. [13] and Kooijman et al. [14] are based on a bone marrow transplantation approach that uses donors with global deficiency of α7nAChR. Whereas this strategy has been successfully used by us [15] and others [19, 31, 32] to evaluate the contribution of a particular gene expressed by macrophages to atherogenesis, it is difficult to conclude whether the reduction in advanced lesion burden and macrophage content in mice with bone marrow deficiency of α7nAChR is the result of the impact of α7nAChR deficiency on macrophage functions or whether cells other than macrophages also contribute to the observed phenotype. Future studies in mice with macrophage specific deletion of α7nAChR will provide more definitive answers to these questions. The recent generation of a mouse model with floxed α7nAChR gene represents a key step towards making mice with conditional deficiency of α7nAChR a reality [33].

The human α7nAChR gene has several single nucleotide polymorphisms. However, clinically associated human variations have not been reported. In expression profile studies using human genomic microarrays and samples from plaques of patients with atherosclerotic lesions in the left anterior descendent coronary artery [34], circulating mononuclear cells from atherosclerotic patients [35] or from plaques of patients with advanced carotid artery disease [36], levels of α7nAChR mRNA are not different from those in control patients. Plaques from patients with advanced carotid artery disease show immunoreactivity for α7nAChR, the majority of which co-localizes with macrophages and T cells [13], but the lack of control staining on non-diseased arteries prevents from conclusions to be made whether this reflects normal levels of α7nAChR protein in these cells or altered expression that accompanies the atherogenic process. It is possible that changes in channel activity and/or its signaling properties, rather than changes in expression level, or alterations in one or more of the signaling partners that complete the α7nAChR signaling pathway, account for differences in α7nAChR functions in physiological *vs*. disease states.

As mentioned above, despite a great deal of evidence documenting the participation of α7nAChR in processes that are relevant to the pathogenesis of atherosclerosis [1, 2, 4, 11, 37], the impact of α7nAChR in either murine or human atherosclerosis has remained largely unexplored. In this context, our findings demonstrating a reduction in size and cellularity of advanced lesions in mice with bone marrow specific deficiency of α7nAChR provide novel experimental evidence underscoring a potential role of macrophage α7nAChR in advanced atherosclerosis and set a framework for future studies aimed at characterizing the mechanisms coupling α7nAChR signaling to macrophage function in atherosclerosis.
